# Supplementary material for: His‐MMDM: Multi‐Domain and Multi‐Omics Translation of Histopathological Images with Diffusion Models
Source: Adv Sci (Weinh). 2026 Jan 26;13(19):e18066. doi: 10.1002/advs.202518066 (PMC13045368; doi:10.1002/advs.202518066)
Supplement: Supplementary file 1 — Supporting File: advs74015‐sup‐0001‐SuppMat.pdf [file ADVS-13-e18066-s001.pdf]

Supporting Information

His-MMDM: Multi-domain and Multi-omics Translation of Histopathological Images with Diffusion Models

Zhongxiao Li[1,2,3,#], Tianqi Su[4,#], Bin Zhang[1,2], Wenkai Han[1,2], Sibin Zhang[4], Guiyin Sun[4], Yuwei Cong[5], Xin Chen[6], Jiping Qi[5], Yujie Wang[4], Shiguang Zhao[6,], Hongxue Meng[7,*], Peng Liang[4,*], and Xin Gao[1,2,*]*

[1]Computer Science Program, Computer, Electrical and Mathematical Sciences and Engineering Division, King Abdullah University of Science and Technology (KAUST), Thuwal, Saudi Arabia

[2]Center of Excellence on Smart Health, King Abdullah University of Science and Technology (KAUST), Thuwal, Saudi Arabia

[3]Department of Life Science, Dezhou University, Dezhou, China

[4]Department of Neurosurgery, Harbin Medical University Cancer Hospital, Harbin, China

[5]Department of Pathology, The First Affiliated Hospital of Harbin Medical University, Harbin, China

[6]Department of Neurosurgery, The First Affiliated Hospital of Harbin Medical University, Harbin, China

[7]Department of Pathology, Harbin Medical University Cancer Hospital, Harbin, China

[#]Equal Contribution

[*]All correspondence should be addressed to Xin Gao (E-mail: xin.gao@kaust.edu.sa, Tel: +966-12-8080323), Shiguang Zhao (E-mail: guangsz@hotmail.com), Hongxue Meng (E-mail: menghongxue@hrbmu.edu.cn), and Peng Liang (E-mail: liangpeng@hrbmu.edu.cn)

Supplementary Text

Section S1 Additional results of IHC virtual staining

Using color deconvolution, we computed the intensity of the DAB stain on the virtually stained images in each glioma and meningioma subtype (**Fig. S2F-S2H**). Most notably, the positivity of IDH-1 (suggesting IDH-1 mutation) is higher in lower-grade glioma subtypes (AA, AO, DA, and O) rather than in glioblastomas (GBM), which is consistent with previous findings that IDH-1 mutations are more frequently found in the lower-grade gliomas or secondary GBMs that stem from them, rather than the primary GBMs [1] (**Fig. S2F**). The intensity of subtype-nonspecific markers tends to correlate with the

proliferation activity and cell density of the specific subtypes. For example, Oligo-2, as a transcription factor necessary in the development of nearly all glioma subtypes [2], tends to have a higher intensity in the more proliferative GBM subtype (**Fig. S2F**). Similarly, EMA[3], PR [4], and SSTR2 [5], being common meningioma markers, tend to have higher intensity in the more proliferative atypical or the more dense fibrous subtype (**Fig. S2G**). As for the common markers used in both glioma and meningioma, CD34, an endothelium marker, reasonably exhibits a higher intensity in angiomatous meningioma and in GBM where angiogenesis is frequent (**Fig. S2H**) [6]. Additionally, GBM and the higher-grade atypical meningioma also show higher intensity of the cell proliferation marker Ki67 (suggesting higher expression) and tumor suppressor p53 (suggesting p53 mutation). Collectively, these analyses demonstrate the effectiveness of His-MMDM in virtual staining and the consistency of the synthesized images with prior knowledge.

<u>Section S2 Additional results of primary tumor type translation</u>

As a comparison, we also trained two GAN-based models, CycleGAN [7] and CUT [8] (**Methods**), and two other diffusion model-based methods D2C [9] (**Methods**). The Frechet Inception Distance (FID) [10] of the translated images to a particular tumor type and the real images of that tumor type were computed as the evaluation of image fidelity (the *lower* FID scores imply *higher* fidelity) (**Methods**). We further computed the FID scores of the real un-translated images between tumor types and computed the reduction of this metric ($\Delta FID = FID_{before} - FID_{after}$) before and after translation by His-MMDM. Overall, His-MMDM achieved similar or even better performance (ΔFID) compared to its GAN-based counterparts, especially for tumor types such as BLCA, KIRP, and LUSC (**Fig. S3A**). His-MMDM also achieved better performance than D2C. The largest ΔFIDs are observed between very distant tumor types, such as between the GYN tumors and kidney tumors (**Fig. S3B**). In particular, due to the distinctiveness of kidney and liver tumors, their ΔFID values are the highest when other tumors are either translated to or from them (**Fig. S3B**). Previous cross-classification studies using discriminative models reported high performance between related tumor types, such as within the GI tumors or the lung tumors [11]. Consistently, we also observed that the ΔFID values tend to be the lowest within the GI tumors and relatively low within the GYN tumors, kidney, and lung tumors.

We compared the performance (in terms of F1 score) of binary tumor classification models trained on one tumor type (the target of translation) and cross-classifying images from another tumor type (the source of translation/the goal of classification) using either the original images or the translated images. The classification performance in four out of five tumor families corresponding to 12 out of 19 tumor types on average showed higher performance using the translated images (**Fig. 3C** and **Fig. S3D**, 'ratio for fine-tuning = 0'). Additionally, to improve the classification models' performance, we fine-tuned the classification models using 30%, 60%, and 90% of the translated images (**Fig. 3C** and **Fig. S3D**, 'ratio for fine-tuning = 0.3, 0.6, 0.9'). The number of tumor types with higher performance using the translated images increases to 13 at 60% and 15 at 90%. The performance improvement is most evident when translating from the GI tumors to the lung tumors, and the improvement is more in recall than in precision (**Fig. S3E**). This improvement in recall could be due to the adaptation of the features of the unknown-type image into known ones. Therefore, translating images from unseen tumor types to known

tumor types is indeed beneficial for a discriminative model's inference and further fine-tuning.

Inference of tumors of unknown primary (TUP) has recently gained substantial attention due to the strong performance of machine learning models on this task, and their demonstrated usefulness in supporting pathologists' clinical decision-making [12-14]. In contrast to previous studies that primarily focused on developing discriminative models, we show that His-MMDM is also effective for interpreting such decisions from a generative modeling perspective. While discriminative approaches typically rely on attention heatmaps or attribution scores to justify their predictions, His-MMDM generates multiple versions of a query image corresponding to all possible tumor origins (**Fig. S4A**). These generated images allow pathologists to visually examine each potential origin, thereby increasing confidence in the determination of the tumor origin. Specifically, for a given histopathology image, we used His-MMDM to translate it into each of the 14 primary tumor sites on which it was trained, computed the cosine distance between the original image and each translated image using Inception V3 features, and selected the translation with the lowest cosine distance as the predicted tumor site (**Fig. S4A**). Across nine of the 14 primary tumor sites, the translation corresponding to the true primary origin yielded the lowest cosine distance (**Fig. S4B**–**S4C**), and in all cases the true origin ranked within the top two categories. These results indicate strong concordance between His-MMDM's interpretative outputs and the true tumor origins. Furthermore, when primary tumor sites were grouped into gastrointestinal (GI), lung (Lung), kidney (Kidney), gynecological (GYN), and other (Others) categories, this interpretative prediction achieved a macro-averaged precision of 0.733 and recall of 0.720 (**Fig. S4D**).

Section S3 Combined genomics and transcriptomics-guided Editing

We finally demonstrate examples when His-MMDM is guided by the combination of mutations and transcriptomic profiles to edit histopathological images. In thyroid tumors, the *BRAF*-*RAS* score is used to distinguish classical thyroid tumors characterized by the $BRAF^{V600E}$ mutation (*BRAF*-like) from the less malignant follicular variant that harbors *RAS* mutations (*RAS*-like). Very recently, a GAN-based generative model has been developed by Dolezal et al. to generate thyroid histopathological images across the full spectrum of the *BRAF*-*RAS* score (ranging from -1 (the most *BRAF*-like) to 1 (the most *RAS*-like)) [15]. One highlight of Dolezal et al.'s model is that once a particular random seed is set, the model can produce *BRAF*-like and *RAS*-like images *that are paired* (see Fig. 3 of Dolezal et al. [15]). We, therefore, investigated whether His-MMDM can recapitulate the patterns of *BRAF*-like and *RAS*-like thyroid tumors learned by the Dolezal et al. model. We first generated paired BRAF-like (score = -1) and RAS-like (score = 1) images using the Dolezal et al. model. Then we used His-MMDM to edit the *BRAF*-like images into *RAS*-like ones and the *RAS*-like images into *BRAF*-like ones, by modifying their mutations (in either *BRAF*, *NRAS*, *HRAS* or *KRAS*) or manipulating their transcriptomic profiles (according to their respective transcriptomic signatures) or both (**Methods**). By comparing the His-MMDM-edited images to the other 'ground truth' image in the pair generated from the Dolezal et al. model, we were able to dissect individual contributions from the genomic side or the transcriptomic side, and the

combined genomic and transcriptomic guidance resulted in higher similarity of the His-MMDM edited images to the 'ground truth' images (**Fig. S9G**). By visualizing the edited images, it is evident that His-MMDM recapitulated the arrangement and the characteristic structural features of thyroid tumors of each subtype, e.g., the papillary structures of the *BRAF*-like and the follicular structures of the *RAS*-like) (**Fig. S9H**).

In colorectal cancer (CRC), the consensus molecular subtypes (CMS) system [16] plays a critical role in patient stratification, diagnosis, and prognosis. The CMS framework integrates transcriptomic data from multiple independent classification schemes and defines four robust subtypes with distinct biological, clinical, and prognostic characteristics. These subtypes capture differences in oncogenic signaling, immune infiltration, metabolic programs, and stromal composition, providing important insights into CRC tumor biology and therapeutic vulnerabilities. Specifically, CMS1 is characterized by hyper–microsatellite instability and strong immune activation; CMS2 is marked by activation of WNT and MYC signaling; CMS3 exhibits epithelial features with prominent metabolic dysregulation; and CMS4 is associated with TGF-β signaling, angiogenesis, and stromal infiltration.

We utilized His-MMDM to encode and re-decode CRC histopathology images according to the somatic mutation profiles and transcriptional dysregulation characteristic of each CMS subtype (**Fig. S10A**). This was achieved through *in silico* knock-up of subtype-specific up-regulated genes represented in the His-MMDM genomic embeddings (n = 71 for CMS1, n = 31 for CMS2, n = 39 for CMS3, and n = 116 for CMS4), together with *in silico* mutation of BRAF in CMS1 tumors and KRAS in CMS3 tumors (**Fig. S10A**). Using a procedure analogous to the tumor-origin prediction task (**Section S2**), we evaluated the cosine-distance–based performance of His-MMDM in predicting CMS subtypes from CRC tumor image patches, achieving macro-averaged precision and recall of 0.717 and 0.706, respectively (**Fig. S10B**).

References

1. Balss, J., et al., *Analysis of the IDH1 codon 132 mutation in brain tumors.* Acta Neuropathol, 2008. **116**(6): p. 597-602.
2. Tsigelny, I.F., et al., *Molecular mechanisms of OLIG2 transcription factor in brain cancer.* Oncotarget, 2016. **7**(33): p. 53074-53101.
3. Petricevic, J., et al., *Expression of nestin, mesothelin and epithelial membrane antigen (EMA) in developing and adult human meninges and meningiomas.* Acta histochemica, 2011. **113**(7): p. 703-711.
4. Maiuri, F., et al., *Progesterone Receptor Expression in Meningiomas: Pathological and Prognostic Implications.* Front Oncol, 2021. **11**: p. 611218.
5. Wu, W., et al., *Clinical Significance of Somatostatin Receptor (SSTR) 2 in Meningioma.* Front Oncol, 2020. **10**: p. 1633.
6. Ahir, B.K., H.H. Engelhard, and S.S. Lakka, *Tumor Development and Angiogenesis in Adult Brain Tumor: Glioblastoma.* Mol Neurobiol, 2020. **57**(5): p. 2461-2478.
7. Zhu, J.-Y., et al. *Unpaired image-to-image translation using cycle-consistent adversarial networks*. in *Proceedings of the IEEE international conference on computer vision*. 2017.

8. Park, T., et al., *Contrastive Learning for Unpaired Image-to-Image Translation*, in *European Conference on Computer Vision*. 2020. p. 319-345.
9. Sinha, A., et al., *D2c: Diffusion-decoding models for few-shot conditional generation.* Advances in Neural Information Processing Systems, 2021. **34**: p. 12533-12548.
10. Heusel, M., et al., *Gans trained by a two time-scale update rule converge to a local nash equilibrium.* Advances in neural information processing systems, 2017. **30**.
11. Noorbakhsh, J., et al., *Deep learning-based cross-classifications reveal conserved spatial behaviors within tumor histological images.* Nature Communications, 2020. **11**(1): p. 6367.
12. Lu, M.Y., et al., *AI-based pathology predicts origins for cancers of unknown primary.* Nature, 2021. **594**(7861): p. 106-110.
13. Ma, W., et al., *New techniques to identify the tissue of origin for cancer of unknown primary in the era of precision medicine: progress and challenges.* Briefings in Bioinformatics, 2024. **25**(2): p. bbae028.
14. Tian, F., et al., *Prediction of tumor origin in cancers of unknown primary origin with cytology-based deep learning.* Nature Medicine, 2024.
15. Dolezal, J.M., et al., *Deep learning generates synthetic cancer histology for explainability and education.* npj Precision Oncology, 2023. **7**(1): p. 49.
16. Guinney, J., et al., *The consensus molecular subtypes of colorectal cancer.* Nature Medicine, 2015. **21**(11): p. 1350-1356.
17. Sanchez-Vega, F., et al., *Oncogenic Signaling Pathways in The Cancer Genome Atlas.* Cell, 2018. **173**(2): p. 321-337.e10.
18. Fu, Y., et al., *Pan-cancer computational histopathology reveals mutations, tumor composition and prognosis.* Nature Cancer, 2020. **1**(8): p. 800-810.
19. Subramanian, A., et al., *Gene set enrichment analysis: a knowledge-based approach for interpreting genome-wide expression profiles.* Proceedings of the National Academy of Sciences, 2005. **102**(43): p. 15545-15550.
20. Liberzon, A., et al., *The Molecular Signatures Database Hallmark Gene Set Collection.* Cell Systems, 2015. **1**(6): p. 417-425.
21. Schmauch, B., et al., *A deep learning model to predict RNA-Seq expression of tumours from whole slide images.* Nature communications, 2020. **11**(1): p. 1-15.

Figure S1. Evaluation of cryosection to FFPE conversion. Related to Figure 2.
(**A**) Evaluation of His-MMDM (in terms of Improved Precision) in performing cryosection to FFPE conversion and comparison with other dedicated (AI-FFPE and its various ablations, AI-FFPE w/o SR, AI-FFPE w/o SAB), non-dedicated GAN-based (CycleGAN, CUT, StyleGAN2), and diffusion model-based (D2C) image translation models. Error bars indicate 95% confidence interval.
(**B**) Evaluation of His-MMDM (in terms of Improved Recall) in performing cryosection to FFPE conversion and comparison with other dedicated (AI-FFPE and its various ablations, AI-FFPE w/o SR, AI-FFPE w/o SAB), non-dedicated GAN-based (CycleGAN, CUT, StyleGAN2), and diffusion model-based (D2C) image translation models. Error bars indicate 95% confidence interval.
(**C**) Example cryosection histopathological images converted by His-MMDM and other baselines.
(**D**) Pathologists rated the quality of His-MMDM–converted cryosectioned images on a 1–5 scale across four criteria: nucleus shape, cell distribution, background contrast, and correction of low-quality regions/artifacts. Each dot indicates the rating of one histopathological image by a particular pathologist (Pathologist 1-4), or the rating averaged across the four pathologists (Avg.). P values were calculated using the Wilcoxon signed-rank test by comparing average pathologist scores to the neutral score of 3.

Figure S2. Cryosection to FFPE conversion and virtual IHC staining of histopathological images. Related to Figure 2.
(**A**) The schematic illustrating the usage of His-MMDM converted cryosectioned slide images for image-to-image retrieval and text-guided classification using the pre-trained multi-modal foundation model, PLIP.
(**B**) His-MMDM-converted cryosectioned slide images improve the text-guided classification accuracy of the PLIP model. FFPE: using FFPE images, Cryo: using original cryosection images, His-MMDM: using His-MMDM translated images.
(**C**) A schematic that illustrates how His-MMDM achieves virtual staining of multiple IHC markers.

(**D**) More examples of virtual staining of glioma images, by both the glioma-specific IHC markers and the common IHC markers.

(**E**) More examples of virtual staining of meningioma images, by both the glioma-specific IHC markers and the common IHC markers.

(**F-H**) The violin plots showing the intensity of the positive (DAB) stain on the virtually-stained images of glioma-specific markers (**F**) meningioma-specific markers (**G**) and common markers (**H**) among the primary brain tumor subtypes.

Figure S3. Translation of histopathological images across primary tumor types. Related to Figure 3.

(**A**) Evaluation of His-MMDM (in terms of ΔFIDscores) in performing cross-tumor type translation and comparison with other image translation models. Error bars indicate 95% confidence interval.

(**B**) Detailed pairwise heatmap showing the improvement of FID score ($\Delta\text{FID} = \text{FID}_{\text{before}} - \text{FID}_{\text{after}}$) between each pair of TCGA classes. Bar plots show the averaged ΔFID for a particular translation source tumor type (rows) or target tumor type (columns).

(**C**) Pathologists rated the quality of His-MMDM–converted images on a 1–5 scale using two criteria: retainment of cellular content and modification of background. Each dot indicates the rating of one histopathological image by a particular pathologist (Pathologist 1-4), or the rating averaged across the four pathologists (Avg.). P values were calculated using the Wilcoxon signed-rank test by comparing average pathologist scores to the neutral score of 3.

(**D**) The classification performance (F1 score) of binary tumor classifiers that are trained on other tumor types (as targets of translation) using either translated/untranslated images when there is no additional fine-tuning ('Ratio for fine-tuning = 0') or with additional fine-tuning ('ratio for fine-tuning = 0.3, 0.6, 0.9') in each tumor type (as the source of translation and the goal of classification). Data for each source of translation is displayed separately. Error bars indicate 95% confidence interval.

(**E**) Improvement of classification performance (F1 score, precision, and recall) of binary tumor classifiers in one tumor type (as the source of translation, rows) that are trained on

other tumor types (as the targets of translation, columns). Improvement is computed as the improvement of the metrics after image translation by His-MMDM.

Figure S4 Translation of histopathological images between tumor types aids tumor origin detection. Related to Figure 3.

(**A**) Schematic showing the process of translating a colon tumor image to multiple different tumor types from different tumor origins, and by evaluating the cosine distance between the translated and original image, His-MMDM can interpretably aid tumor origin detection.

(**B**) Example interpretations of TUP prediction models by translating query images into different primary sites. The cosine distances of the respective translated images to the original query image are provided along with the translated ones. The correct class is marked in blue.

(**C**) Violin plots showing the distribution of the cosine distances between the translated images and the original query images, grouped by the categories of the query image.

(**D**) Per-class and macro-averaged precision and recall metrics for the five major tumor types in the TCGA dataset.

Figure S5 Translation of histopathological images between primary and metastatic organ sites. Related to Figure 4.

(**A**) A schematic that illustrates how His-MMDM achieves histopathological image translation between different primary organ sites.

(**B**) Pathologists rated the quality of His-MMDM–converted images on a 1–5 scale using two criteria: retention of cellular content and modification of background. Each dot indicates the rating of one histopathological image by a particular pathologist (Pathologist 1-4), or the rating averaged across the four pathologists (Avg.). P values were calculated using the Wilcoxon signed-rank test by comparing average pathologist scores to the neutral score of 3.

(**C**) Visualization of running the outlier detection model (Isolation Forest) on the translated images from the lung to the brain. The images are visualized according to their structure in the tSNE space, and their ‘inlier scores’ are superimposed.

(**D**) Pathologist-assessed visual quality on randomly selected translated images. For the images translated to lymph node and brain, 20 images were randomly selected from the inliers (in the interior of the tSNE grid), and outliers (on the edge of the tSNE grid), and a pathologist ranked the visual quality of each of them (on a 1-5 scale).

(**E**) The associations of other clinical indicators with TCGA patients' overall survival.

(**F**) The p-values from the Cox model of the previous associations compared to the lymph node and brain inlier scores.

Figure S6 The genomics-guided editing of histopathological images. Related to Figure 5.

(**A**) Example COAD images edited into different single mutations (*APC*, *TP53*, *SMAD4*, *DCHS2*, and *NF1*). Structural distance maps are shown to highlight the differences.

(**B**) The cosine distance (InceptionV3 feature) and cosine distance (CHIEF feature) between edited images have a very high correlation (Pearson $r = 0.95$, Spearman $\rho = 0.98$).

(**C**) The cosine distance (InceptionV3 feature) and structural distance between edited images have a high correlation (Pearson $r = 0.78$, Spearman $\rho = 0.98$).

(**D**) The effect of mutations on histopathological images by each tumor type, measured by the cosine distance (CHIEF feature) between the WT version image and the mutated image.

(**E**) The effect of mutations on histopathological images by each tumor type, measured by the structural distance between the WT version image and the mutated image.

(**F**) Performance of the first pathologist's recognition of the top mutations in four tumors (COAD, UCEC, OV, and ESCA) before and after observing His-MMDM's generated images as a tutorial. Improvement is observed except for TP53 in OV.

(**G**) Performance of the second pathologist's recognition of the top mutations in four tumors (COAD, UCEC, OV, and ESCA) before and after observing His-MMDM's generated images as a tutorial. Improvement is observed except for TP53 in OV.

(**H**) Performance of pathologists' recognition of the top mutations in four tumors (COAD, UCEC, OV, and ESCA) before and after observing real, unpaired images as a tutorial. In the left four panels, statistical significance of improvement is calculated base

on simulating a null distribution of the percentage of correct answers when the pathologists' decisions are random. In the rightmost panel, the results from different tumor type-mutation pairs are pooled together, and the p-value from the Wilcoxon rank sum test is shown.

(**I**) Performance (f1 score) of a binary classification model's recognition of the top mutations in four tumors (COAD, UCEC, OV, and ESCA) after being trained on His-MMDM-generated images. Experiments were conducted at four different train set sizes (1k, 2k, 3k, and 4k) and repeated three times. Error bars indicate 95% confidence interval.

Figure S7 The genomics-guided editing of histopathological images, accumulating mutations. Related to Figure 5.

(**A**) Cosine distances of images edited with sequentially accumulating mutations to the WT version of the image. The images sequentially (sorted by mutation rate from high to low) performed the mutation of 25%, 50%, 75%, and 100% of genes in each oncological pathway. The cosine distances were computed w.r.t. the WT version (0% mutation) and visualized through each line plot. Each line plot is colored with the median cosine distance (cross-tumor normalized) when 25%, 50%, 75%, and 100% were mutated. Bar plots aggregate per each pathway (row) or tumor type (row).

(**B**) Example COAD images edited into accumulating mutations (0%, 25%, 50%, 75%, and 100% of genes, ordered by mutation rate) of four different oncological pathways (Cell Cycle, WNT, TP53, and RTK-RAS).

Figure S8 The transcriptomics-guided editing of histopathological images. Related to Figure 6.

(**A**) The effect of transcriptional pathway manipulations on histopathological images by each tumor type, measured by the cosine distance (CHIEF feature) between the WT version image and the mutated image.

(**B**) The effect of transcriptional pathway manipulations on histopathological images by each tumor type, measured by the structural distance between the WT version image and the mutated image.

(**C**) Examples of MSigDB transcriptomic pathway manipulations.

(**D**) The computation of the change in cosine distance of a query image with a certain pathway down-regulated to its nearest neighbors in the database when the image is edited into a version with that pathway is up-regulated. 20 out of the top 25 tumor type and pathway pairs where His-MMDM's edits resulted in the most notable feature changes displayed a statistically significant reduction in the distance to their nearest neighbors. q values are from the Wilcoxon signed-rank test adjusted by the Benjamini–Hochberg procedure.
(**E**) Performance of pathologists' recognition of the top pathway alterations in four tumors (COAD, UCEC, OV, and ESCA) before and after observing His-MMDM-generated, paired images as a tutorial. In the left four panels, statistical significance of improvement is calculated based on simulating a null distribution of the percentage of correct answers when the pathologists' decisions are random. In the rightmost panel, the results from different tumor type-mutation pairs are pooled together, and the p-value from the Wilcoxon signed-rank test is shown.
(**F**) Performance of pathologists' recognition of the top pathway alterations in four tumors (COAD, UCEC, OV, and ESCA) before and after observing real, unpaired images as a tutorial. In the left four panels, statistical significance of improvement is calculated based on simulating a null distribution of the percentage of correct answers when the pathologists' decisions are random. In the rightmost panel, the results from different tumor type-mutation pairs are pooled together, and the p-value from the Wilcoxon signed-rank test is shown.

Figure S9 Additional transcriptomics-guided editing experiments of histopathological images. Related to Figure 6.
(**A**) Performance (f1 score) of a binary classification model's recognition of the top pathway alterations in four tumors (COAD, UCEC, OV, and ESCA) after being trained on His-MMDM-generated images. Experiments were conducted at four different train set sizes (1k, 2k, 3k, and 4k) and repeated three times. Error bars indicate 95% confidence interval.
(**B**) The complete tumor types in which the knock-up of the pathway 'G2M checkpoint' reduces the number of malignant cells detected by Hover-Net.

(**C**) The complete tumor types in which knock-up of the pathway 'DNA repair' reduces the number of malignant cells detected by Hover-Net.

(**D**) Examples of a KIRC image and a COAD image edited into somatic copy number amplification of different genes.

(**E**) The complete list of translations between immune subtypes that increase the count of necrotic cells detected by Hover-Net. Each dot represents a particular image before (blue) or after (red) translation. P values from the Wilcoxon signed rank test.

(**F**) The same set of translations between the immune subtypes and in the tumor types as (**E**), but performed with randomly sampled genes. Each dot represents a particular image before (blue) or after (red) translation. Almost all translations showed insignificant changes in the count of necrotic cells before and after translation (except LUSC C1→C3 and PRAD C2→C3 showed some levels of significance). P values from the Wilcoxon signed rank test.

(**G**) Translation of THCA images from BRAF-like to RAS-like and vice versa. Cosine distances of the edited THCA images using either genomics alone (G), transcriptomics alone (T), or both (GT) to the 'ground truth' images of Dolezal et al.'s model are reported. When translating in the direction from BRAF-like to RAS-like, there are three options for mutations (HRAS, KRAS, and NRAS), compared to the only option (BRAF) in the opposite direction. Boxplots represent median ± IQR; tails: min/max excluding outliers (±1.5xIQR).

(**H**) Histopathological image examples illustrating the effect of editing images from BRAF-like to RAS-like and vice versa using either genomics alone, transcriptomics alone, or both.

Figure S10 Translation of histopathological images between colon tumor consensus molecular subtypes. Related to Figure 6.

(**A**) Schematic showing the process of translating a colon tumor image to multiple different consensus molecular subtypes (CMS), by manipulating the transcriptional expression levels/mutation status of relevant genes. Then, by evaluating the cosine distance between the translated and original images, His-MMDM can interpretably aid CMS subtype classification.

(**B**) Per-class and macro-averaged precision and recall metrics for the four CMS in the TCGA dataset.

(**C**) The selection of sampling strategies on TCGA, HMU-C, and HMU-1st datasets. The shaded area around the line represents the 95% confidence interval.

Table S1 The model architecture, hyperparameters, training configurations, and task specifications.

(**A**) The detailed architecture of the U-Net model in His-MMDM

(**B**) The hyperparameters used for training and testing

(**C**) Summary of the image translation tasks for His-MMDM

Table S2 Dataset statistics.

(**A**-**C**) Statistics of the TCGA dataset (**A**), the HMU-C dataset (**B**), and the HMU-1st dataset (**C**)

Table S3 Abbreviations of tumor types.

Table S4 Comparison of His-MMDM to original and ablated versions of AI-FFPE on the cryosection to FFPE translation task.

Table S5 Description of the IHC markers used in virtual staining.

Table S6 Relevant genes and pathways used in the experiments.

(**A**) The genes used in the genomic embeddings of His-MMDM. Each gene is marked for (1) its membership in oncological pathways defined in Sanchez-Vega et al. [17] (2) the TCGA cohorts in which they have high mutation rates (3) high discriminative performance reported by Fu et al. [18]. The genes are sorted according to their pan-cancer mutation rate in TCGA.

(**B**) The genes used in the transcriptomic embeddings of His-MMDM. Each gene is marked for (1) its membership in the MSigDB pathways [19, 20] (2) whether they are highly dysregulated in TCGA tumor samples (t-statistic) (3) its membership in the well-predicted gene signatures of HE2RNA [21].

(**C**) The component genes of the MSigDB pathways.

(**D**) The definition of the immune subtypes (C1, C2, and C3) in terms of their expression signatures of five immunological pathways.

(**E**) The component genes of the immunological pathways.

(**F**) The randomly selected genes for the immunological pathways as a negative control.

(**G**) The genes with mutations (genomics) and the genes up-regulated (transcriptomics) in the *BRAF*-like and *RAS*-like thyroid tumors.

Data S1 Pathologist evaluation material for cryosection to FFPE translation (separate file)

Data S2 Pathologist evaluation material for primary tumor type translation (separate file)

Data S3 Pathologist evaluation material for tumor organ site translation (separate file)

Data S4 Pathologist training material (His-MMDM generated, paired) and test material for mutations (separate file)

Data S5 Pathologist training material (His-MMDM generated, paired) and test material for transcriptional alterations (separate file)

Data S6 Pathologist training material (real, unpaired) and test material for mutations (separate file)

Data S7 Pathologist training material (real, unpaired) and test material for transcriptional alterations (separate file)

Figure S1

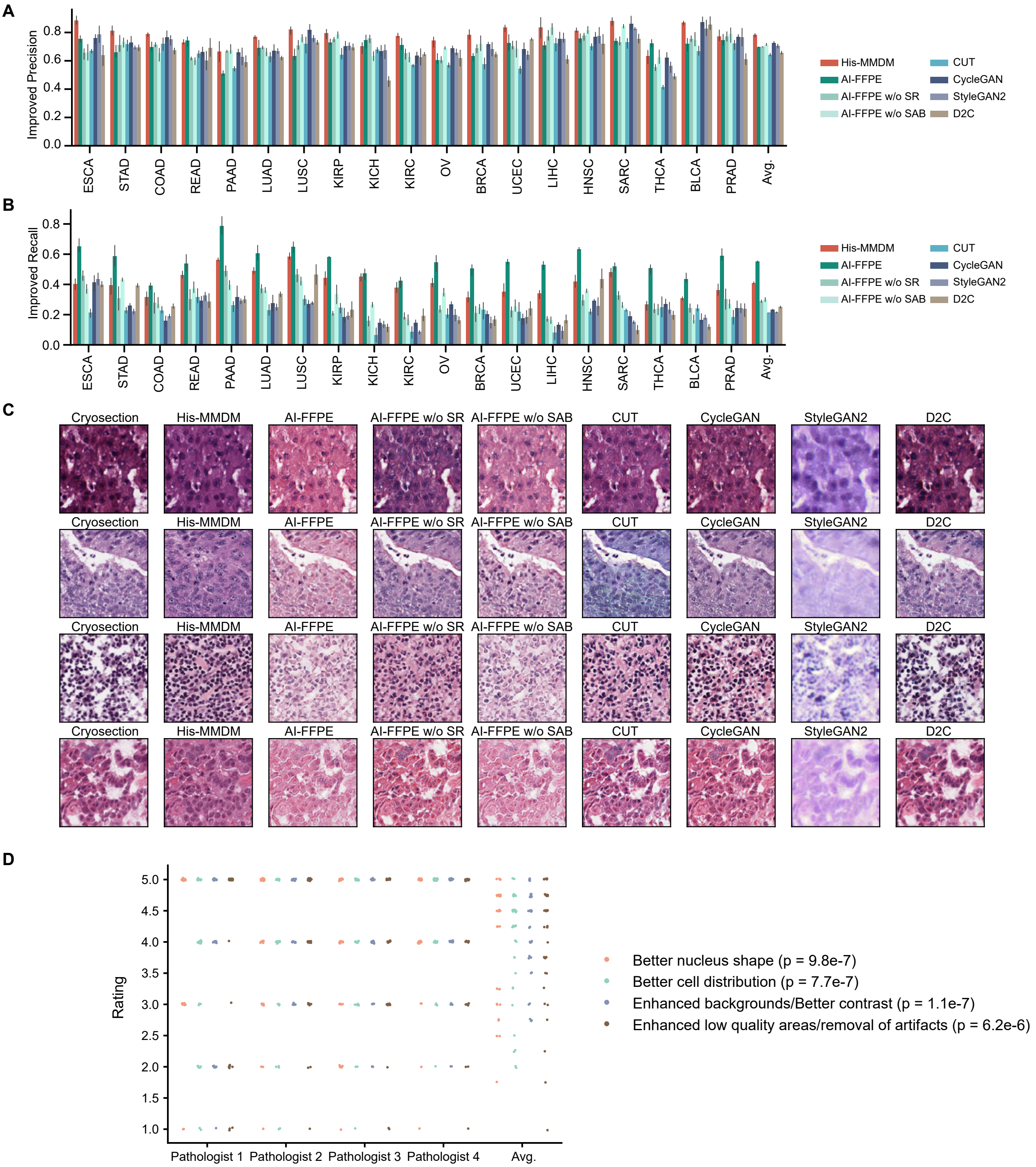

Figure S1

Figure S2

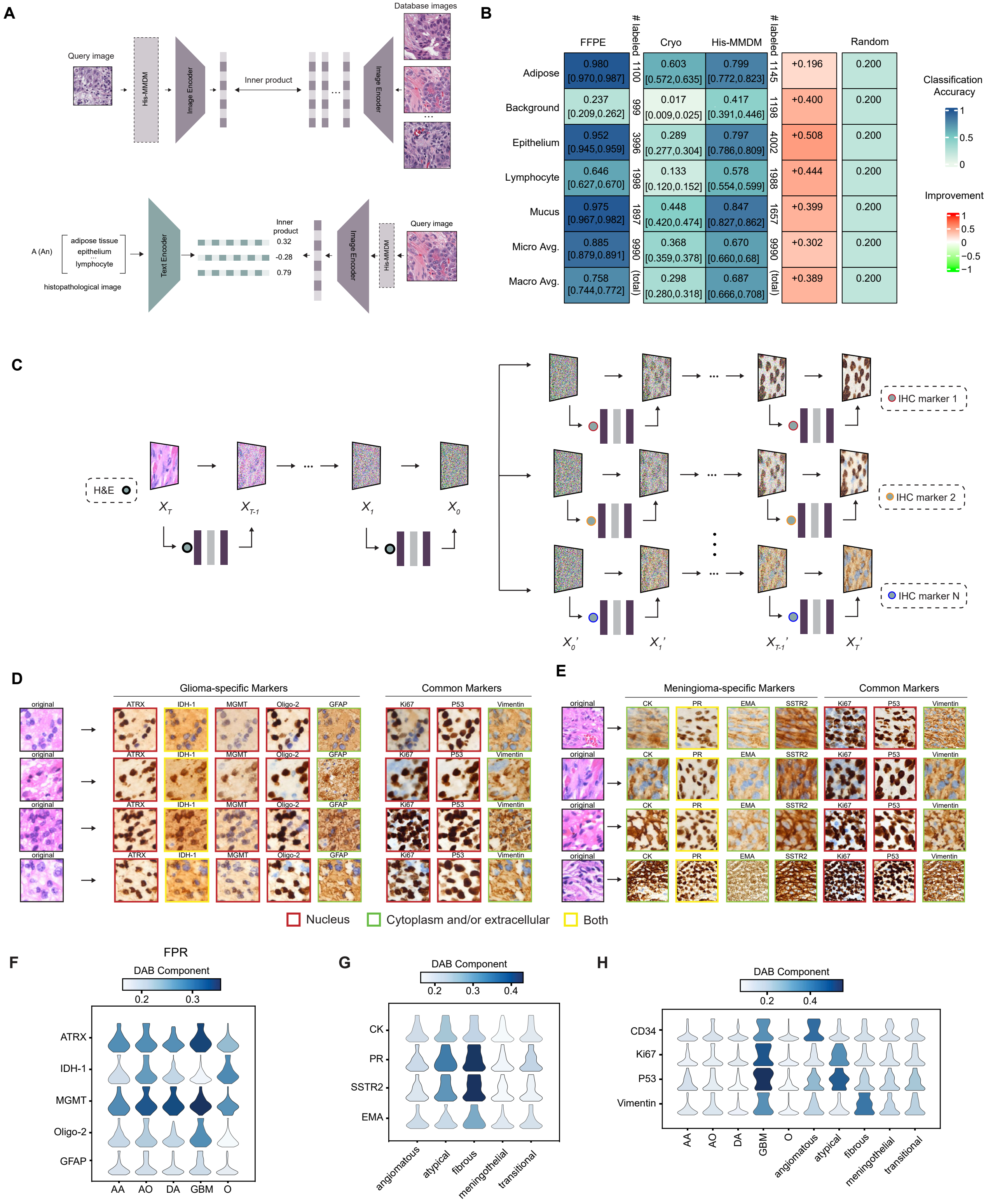

Figure S2

Figure S3

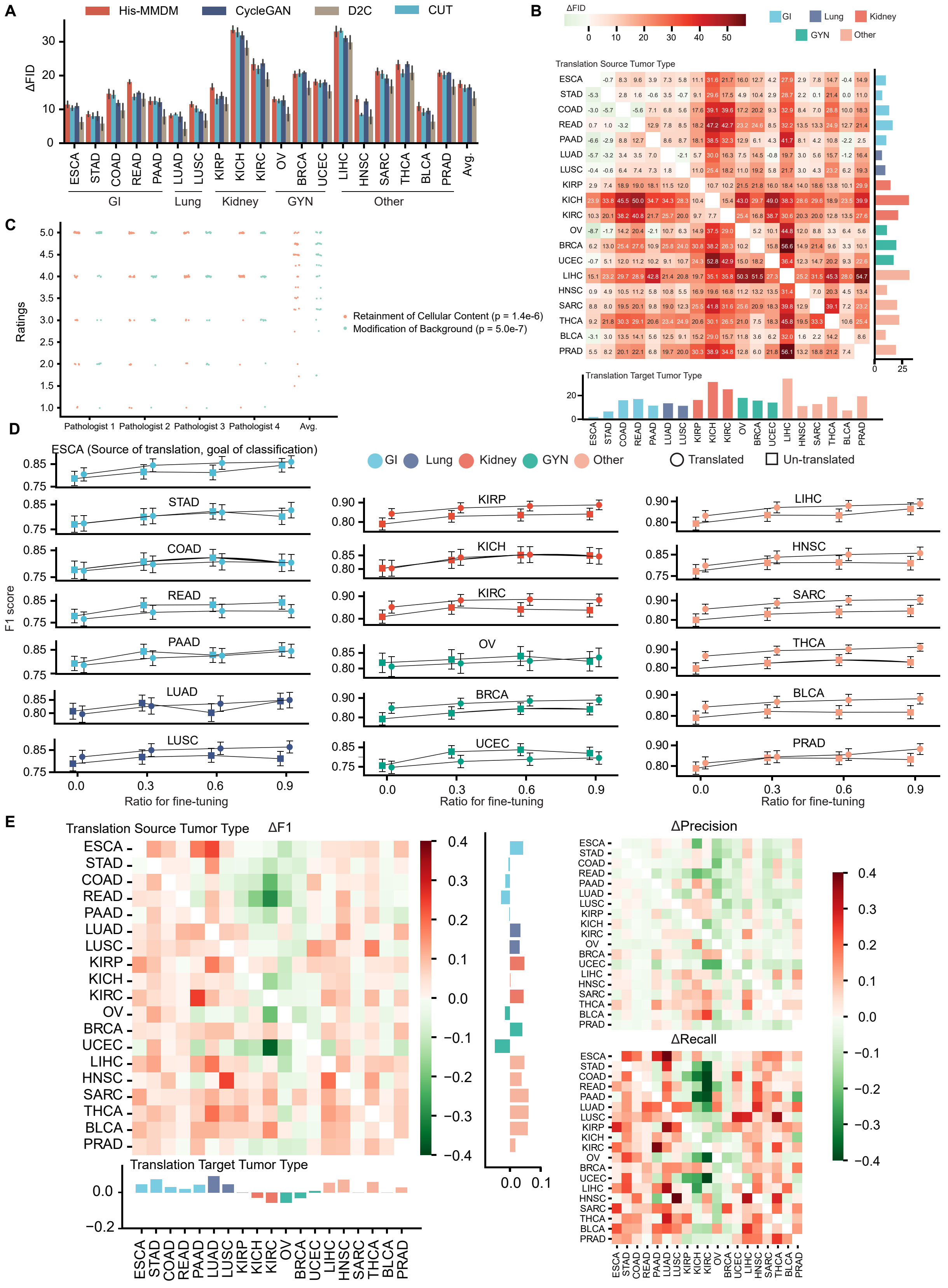

Figure S3

Figure S4

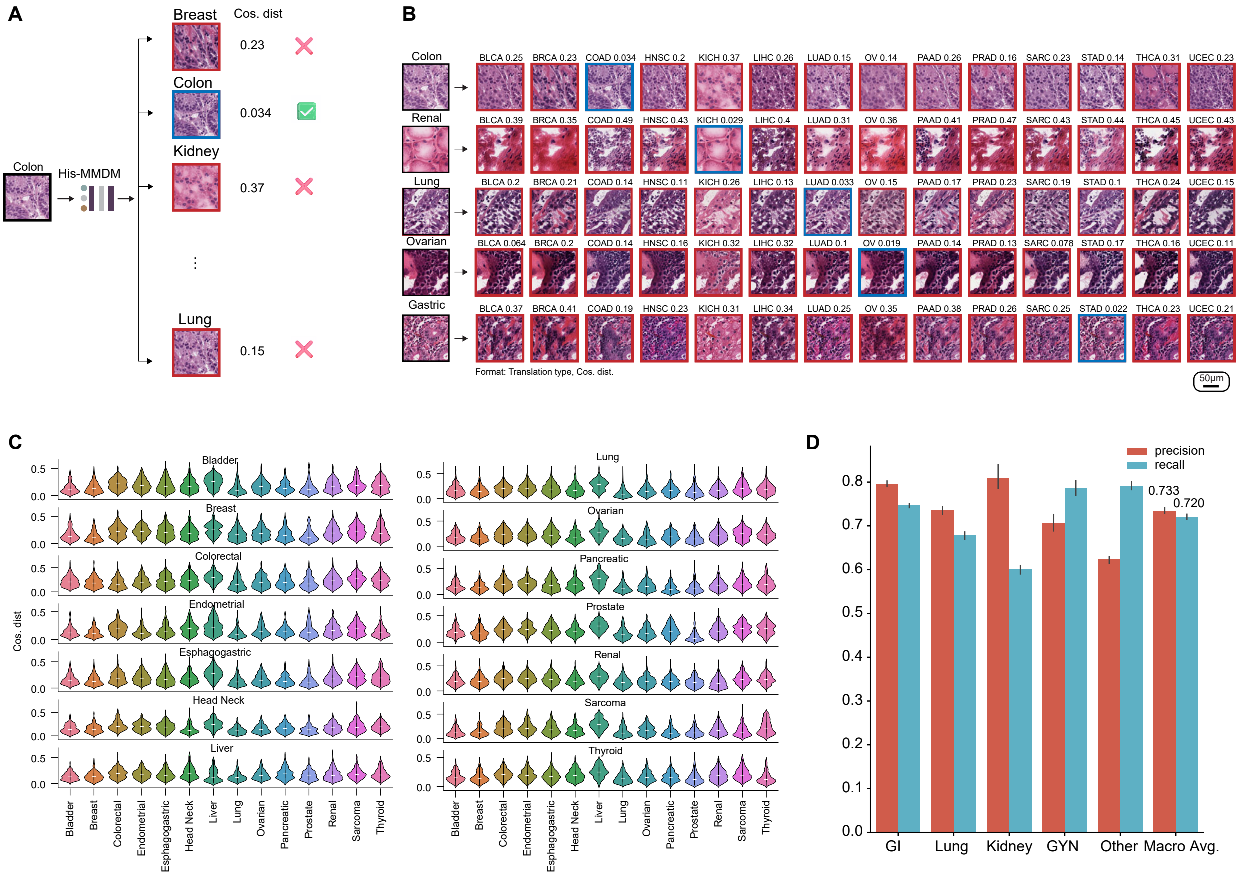

Figure S4

Figure S5

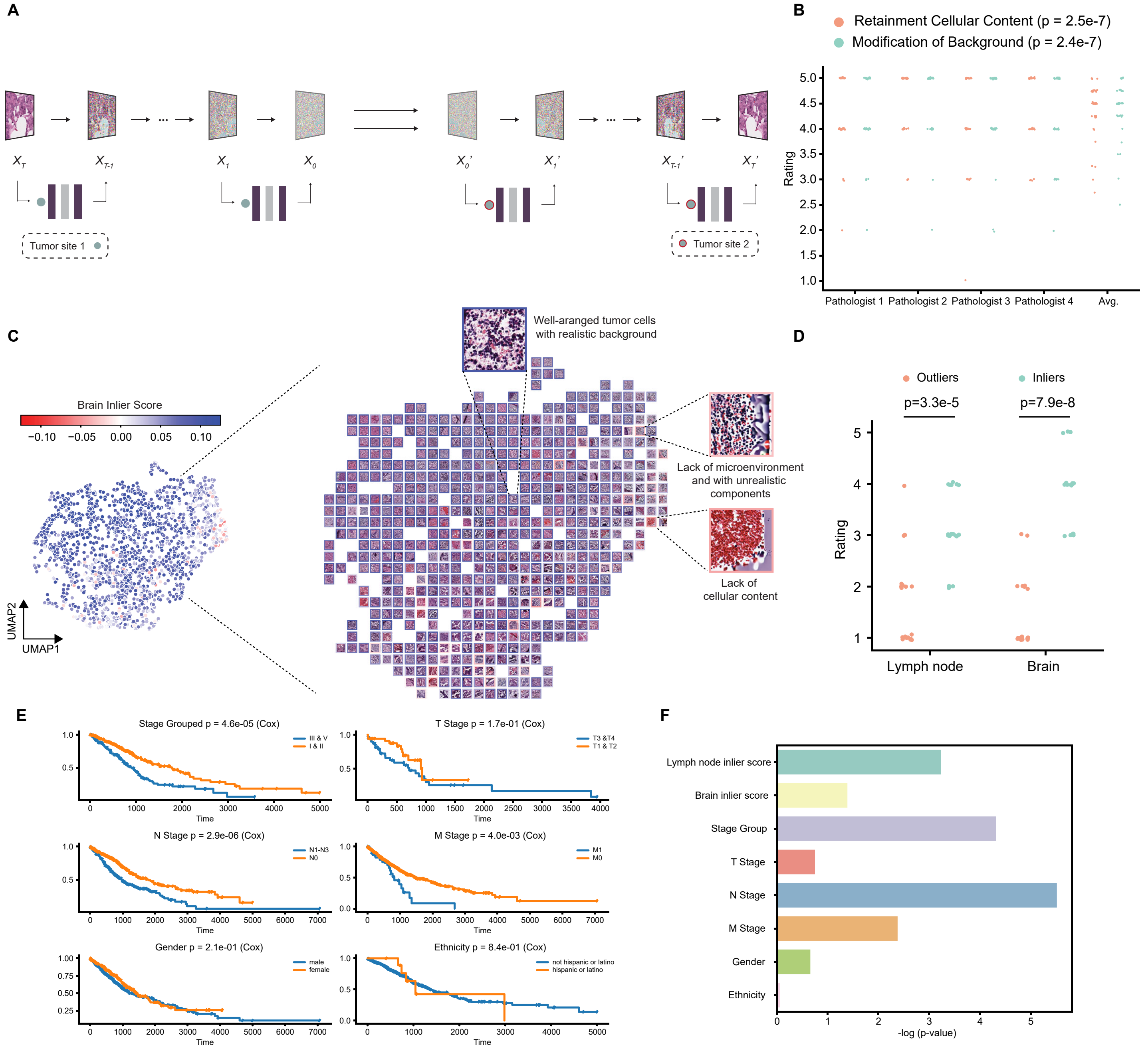

Figure S5

Figure S6

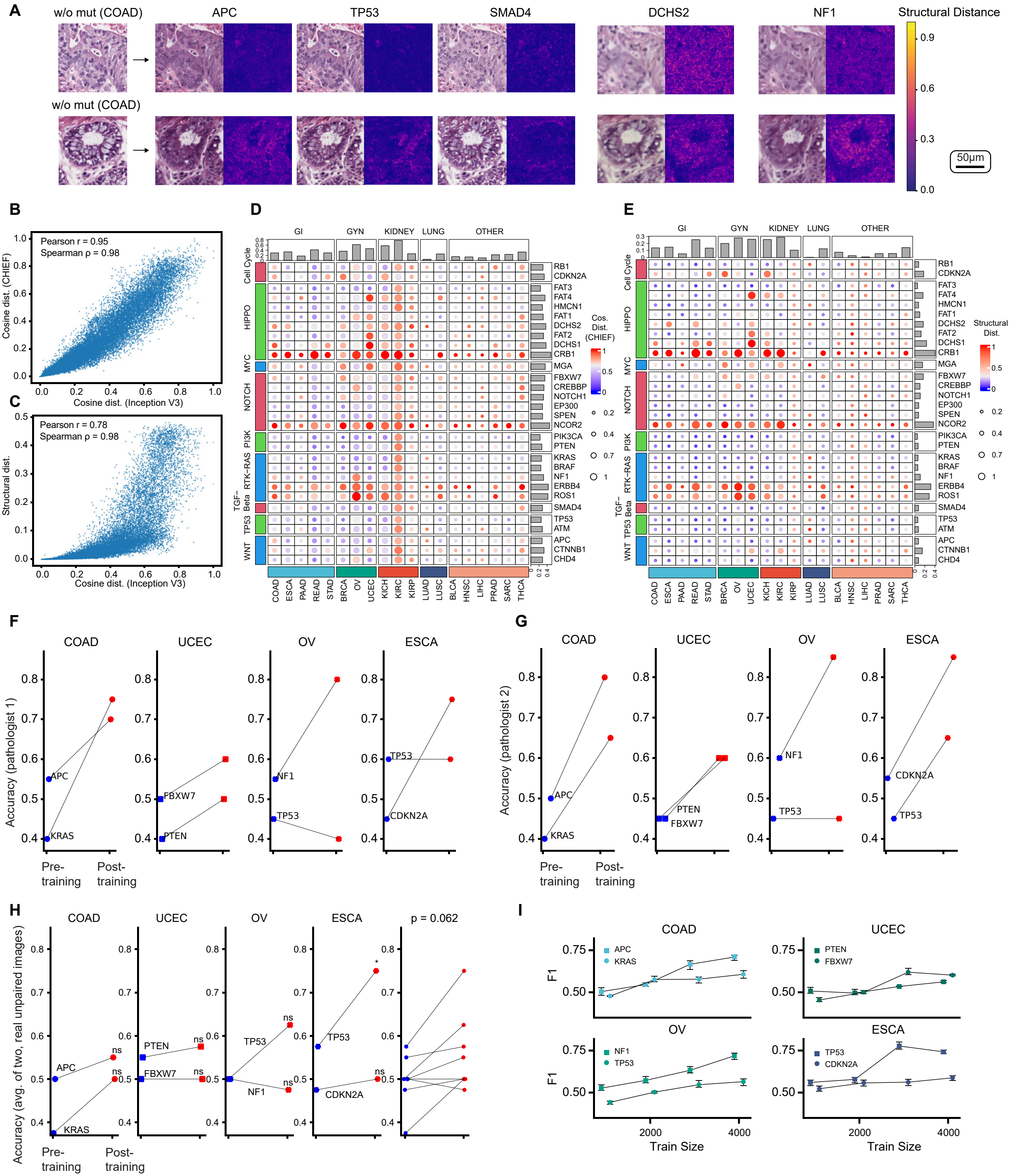

Figure S6

Figure S7

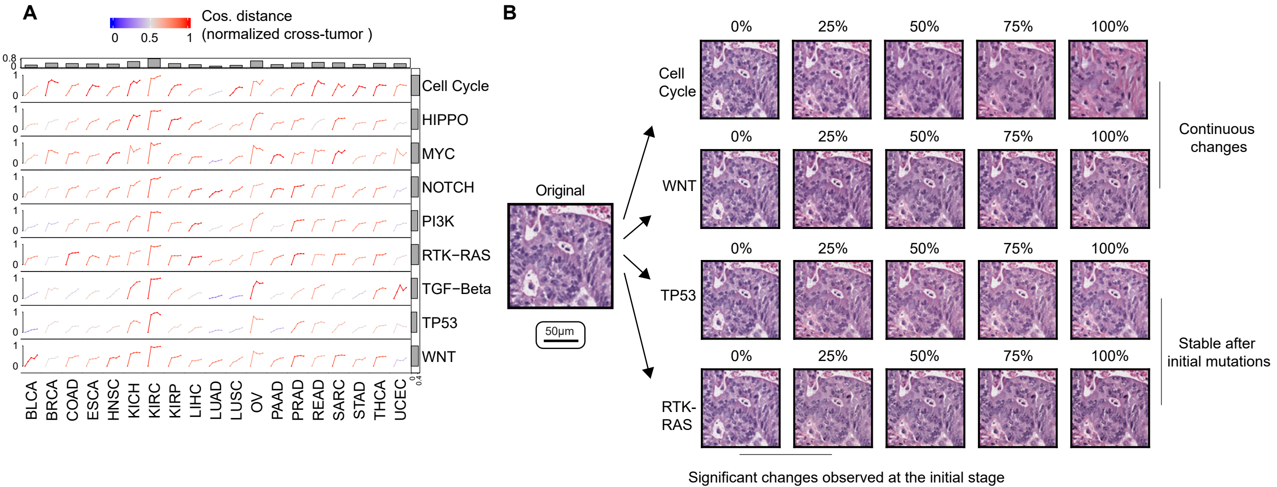

Figure S7

Figure S8

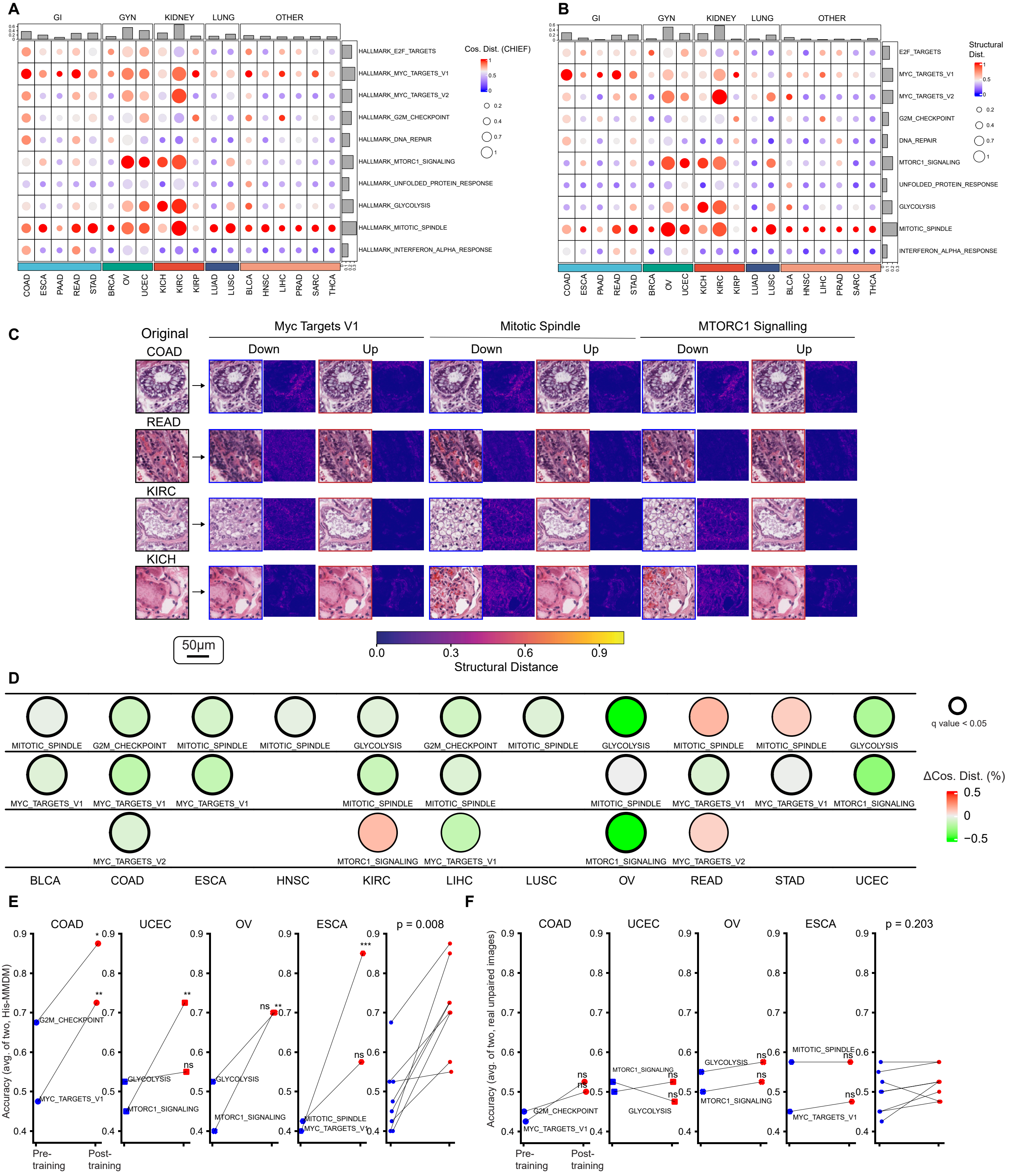

Figure S8

Figure S9

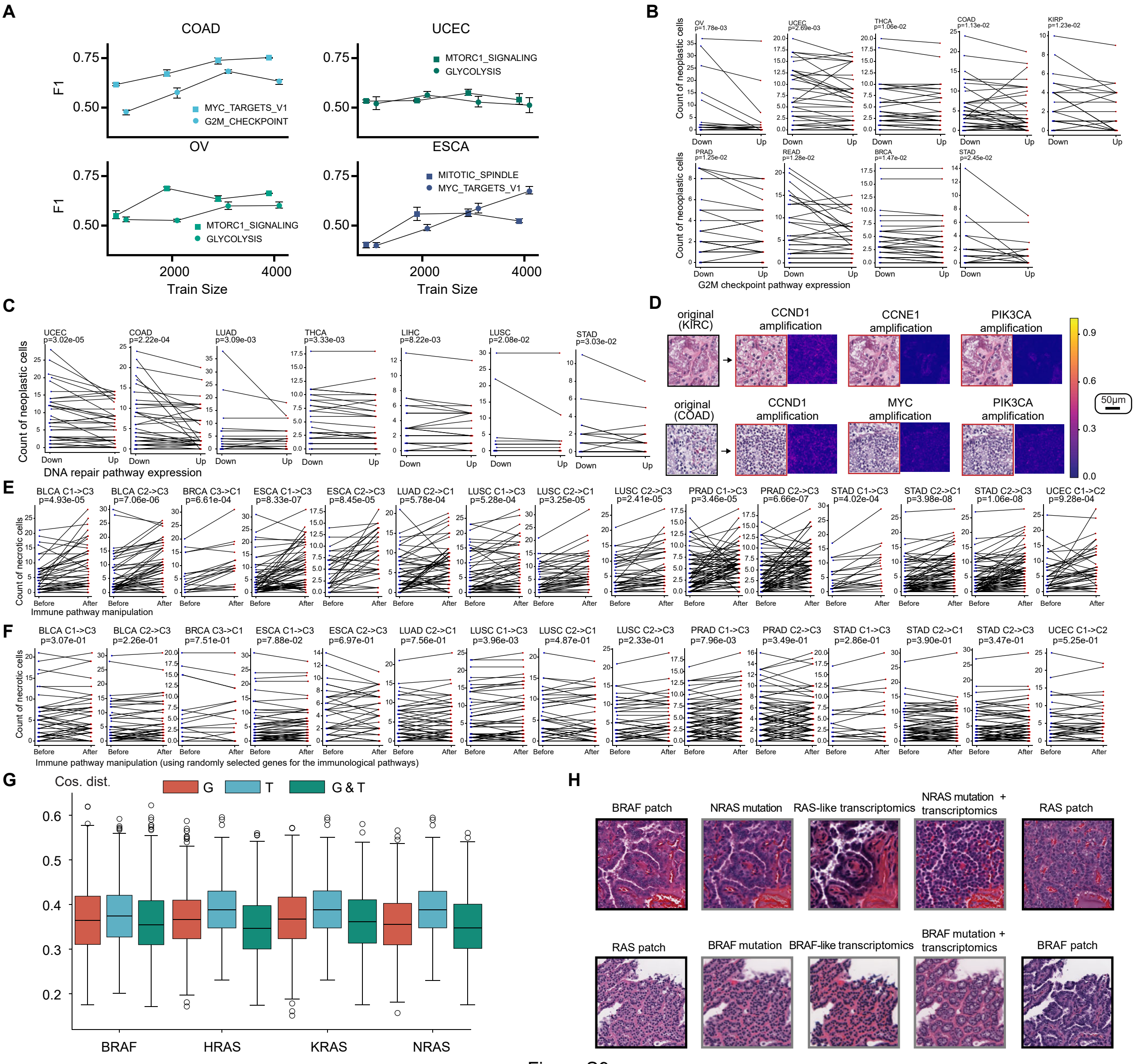

Figure S9

Figure S10

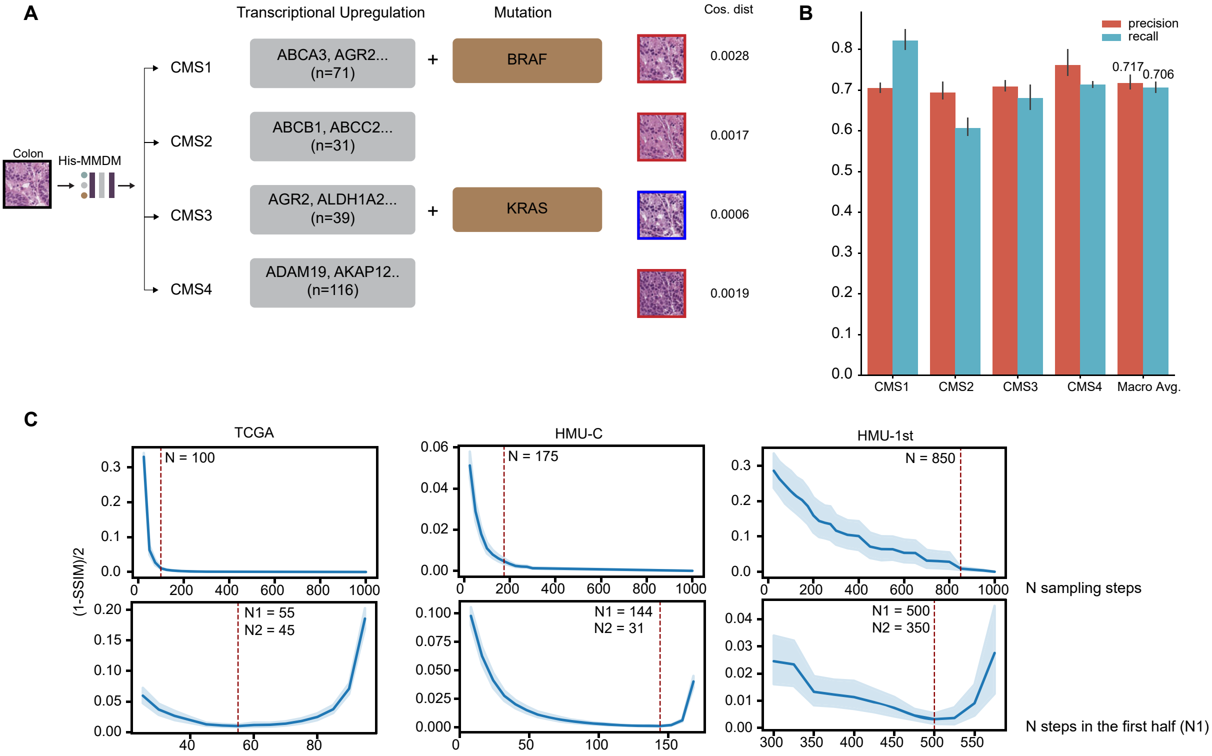

Figure S10
